# Protein Adductomics: Methodologies for Untargeted Screening of Adducts to Serum Albumin and Hemoglobin in Human Blood Samples

**DOI:** 10.3390/ht8010006

**Published:** 2019-03-08

**Authors:** Henrik Carlsson, Stephen M. Rappaport, Margareta Törnqvist

**Affiliations:** 1 Division of Environmental Health Sciences, School of Public Health, University of California, Berkeley, CA 94720, USA; srappaport@berkeley.edu; 2 Department of Environmental Science and Analytical Chemistry, Stockholm University, SE-106 91 Stockholm, Sweden; Margareta.Tornqvist@aces.su.se

**Keywords:** proteins, protein adducts, electrophiles, adductomics, mass spectrometry, hemoglobin, human serum albumin

## Abstract

The reaction products of electrophiles in vivo can be measured as adducts to the abundant proteins, hemoglobin (Hb), and human serum albumin (HSA), in human blood samples. During the last decade, methods for untargeted screening of such adducts, called “adductomics”, have used liquid chromatography-mass spectrometry to detect large numbers of previously unknown Hb and HSA adducts. This review presents methodologies that were developed and used in our laboratories for Hb and HSA adductomics, respectively. We discuss critical aspects regarding choice of target protein, sample preparation, mass spectrometry, data evaluation, and strategies for identification of detected unknown adducts. With this review we give an overview of these two methodologies used for protein adductomics and the precursor electrophiles that have been elucidated from the adducts.

## 1. Introduction

Electrophilic compounds occurring in vivo are generally formed by metabolism of molecules arising from endogenous (e.g., oxidative stress) and exogenous (e.g., diet and drugs) sources. Since these reactive species can modify nucleophilic sites of DNA and functional proteins, they can have toxicological implications. The inherent reactivity of electrophiles makes them difficult to measure in vivo due to their short lifetimes, which has initiated analytical developments to measure their adducts to biomacromolecules. Thus, since the 1970s researchers have studied adducts of reactive electrophiles with abundant blood proteins, to obtain information about cumulative exposures to these reactive species over the mean residence times of particular proteins [1,2,3]. Protein adducts are covalent modifications resulting from reactions between electrophiles and nucleophilic sites in proteins, such as the N-terminus or the amino acid side chains containing sulfhydryl or amine functionalities.

Since the early days of adduct measurements, mass spectrometry (MS) has been the analytical detection technique of choice for the adduct measurements because it provides both qualitative and quantitative information about the modified proteins. Measurements were targeted on adducts from expected reactive metabolites of toxic chemicals with a given protein. Important examples include hemoglobin (Hb) adducts from ethylene oxide [4,5], aromatic amines [6,7], and acrylamide [8,9] in occupational exposed workers and in smokers. Well-known examples of adducts measured to human serum albumin (HSA) are adducts of benzene and styrene in factory workers [10,11]. Many studies also showed a background adduct level in non-exposed control persons, as exemplified with the Hb adducts from acrylamide, shown to be associated with consumption of cooked foods [12], and HSA adducts of aflatoxin B1 from consumption of mycotoxin-contaminated foods [13,14]. These early studies clearly showed that humans have a continuous background exposure to electrophiles, both from endogenous and exogenous sources.

During the last decade there has been a transition to untargeted approaches, “adductomics”, for analysis of the totality of adducts bound to a particular nucleophilic site [15] with the aim to contribute to the characterization of the human exposome [16]. The adductomics concept was first described by Kanaly et al. in 2006, who reported the simultaneous LC-MS detection of numerous DNA adducts in human tissues [17]. A few years later, similar LC-MS approaches were described for protein adductomics by Li et al. [18] focusing on Cys34 adducts of HSA [15] and by Carlsson et al. [19] using N-terminal valine adducts of Hb [20]. These latter two groups, at the University of California, Berkeley and Stockholm University, have continued to pursue adductomics of HSA and Hb, respectively. This review compares the two methodologies and lists the electrophilic species that have been characterized. For an updated and comprehensive overview of DNA adductomics the recent review by Villalta and Balbo is recommended [21].

## 2. Choice of Target Proteins in Blood

Most electrophiles occurring in vivo are generated from enzymatic biotransformations of precursor molecules as well as from reactive oxygen and carbonyl species. Reactive functional groups make electrophiles capable of reacting with nucleophilic loci of proteins, notably, oxygen radicals, epoxides (adducts formed via nucleophilic substitution), activated double bonds of α,β-unsaturated carbonyl compounds (adducts formed via Michael addition), and aldehydes (adducts are Schiff bases formed via carbinolamine intermediates) [22,23]. Important nucleophilic sites for adduct formation in proteins are the sulfhydryl group of cysteine and amine functionalities of histidine, lysine, and N-terminal amino acids [23]. Factors that affect the extent of adduct formation are the nucleophilicity and pKa of the nucleophile and steric factors that influence the access of the reactive electrophile to the nucleophilic site. Nucleophilic sites that are deprotonated at physiological pH (7.4) are favored for adduction because they have free electron pairs that can participate in the formation of covalent bonds. Because the chemistry of adduct formation differs across nucleophilic sites, for example Schiff bases are formed by reactions of aldehydes with amines and the formation of disulfide adducts require free sulfhydryl groups, it would be optimal to include several nucleophilic sites in characterizing a protein adductome.

Important properties of Hb and HSA are summarized in Table 1. Regarding HSA, Cys34 has been the focus of all published studies on HSA adductomics thus far. This cysteine accounts for the antioxidant and detoxifying properties of albumin in the interstitial space and, therefore, is highly conserved in all mammalian species [24,25]. Because Cys34 has an unusually low pK_a_ of 6.55, compared to 8.0–8.55 for most protein thiols [25,26], the sulfhydryl group is predominantly in the deprotonated thiolate form (S^-^) at physiological pH (7.4). Since thiolate is an unusually strong nucleophile, many small electrophiles react preferentially at this site and makes Cys34 an exceptional nucleophilic locus for adductomics [15].

With a few exceptions the in vivo formation of adducts involves only a very small fraction of the total amount of protein. For example, levels of typical Hb adducts of N-terminal valine (30–150 pmol/g Hb) correspond to 5–25 adducted Hb chains per 10^7^ Hb chains in a human blood sample, whereas HSA adduct levels to Cys34 of 0.5–50 nmol/g HSA correspond to 3 modified HSA molecules per 10^3^–10^5^ HSA molecules. Furthermore, since blood is the only tissue that is routinely available in studies of human health, adduct measurements have thus far focused on the two most abundant proteins in blood, i.e., Hb and HSA. The concentrations of Hb and HSA in human blood are approximately 120–160 mg/mL and 35–55 mg/mL, respectively, which enable adductomic analysis using between 5 (HSA) and 250 µL (Hb) of blood per subject. In contrast, the concentration of DNA in human blood is about 30 µg/mL, and DNA adductomics requires about 10 mL blood [27] per subject.

Another important factor is the life-time of the protein in the human body, which provides a time window for integration of chronic exposures to reactive species [23,28]. Human serum albumin has a half-life of approximately 20 days [24] and Hb has a life-time (the same as the erythrocytes) of approximately 120 days [29]. Chemically stable adducts of these proteins accumulate during chronic exposure and such adducts of HSA and Hb reach steady-state levels corresponding to the exposure during about one and two months, respectively [23,28]. This “signal-averaging” reduces variability due to short-term variation in exposure to reactive species and provides more stable measures of long-term exposure for epidemiologic investigations [30]. In contrast, DNA adducts are more variable measures of exposure because of complex kinetics and turnover rates due to enzymatic repair of adducts, and result in low levels of accumulation [23,31].

The adductomics of Hb has focused exclusively on adducts to the N-terminal Val, which has a pK_a_ of approximately 7.8 (α-chain) and 6.8 (β-chain) [32]; Hb is a tetramer composed of two α- and two β-chains, both with Val as the N-terminal amino acid. Another nucleophilic amino acid in Hb of potential interest for adductomic studies is β-Cys93, which is a known adduction site for aromatic amines [7,33]. An important difference between HSA and Hb is the localization of the two proteins; HSA is, except in blood, also found in the interstitial fluid whereas Hb is only found in red blood cells (RBCs). Their chemical environments are therefore partly different, which could give effects on the adductomes observed. In RBCs, the concentration of glutathione (GSH) is high compared to plasma concentrations (about 250 times higher in RBCs [34]), meaning that GSH has a far more important role as an electrophile scavenger in RBCs, giving some protection of Hb from incoming electrophiles. In plasma, Cys34 of HSA has a similar function. The sulfhydryl of Cys34 is a stronger and larger nucleophile than the amino group of N-terminal Hb Val, also with a lower pK_a_, meaning that higher adduct levels are to be expected to this nucleophilic site. This is confirmed when comparing adduct levels from a few electrophilic precursors in the published adductomics studies, with Hb adduct levels typically in the pmol/g Hb range and HSA adduct levels in the nmol/g HSA range. In fact, the human in vivo reaction rate between benzene oxide and HSA Cys34 was 30 times greater than that for Hb β-Cys93 [35]. This also means that much lower sample volumes may be used for analysis of HSA adducts compared to Hb adducts.

## 3. Sample Preparation

In this section we describe and compare the sample preparation procedures for HSA and Hb adductomics [36,37]. Critical differences between the two methodologies are summarized in Table 2.

**Figure 1:**
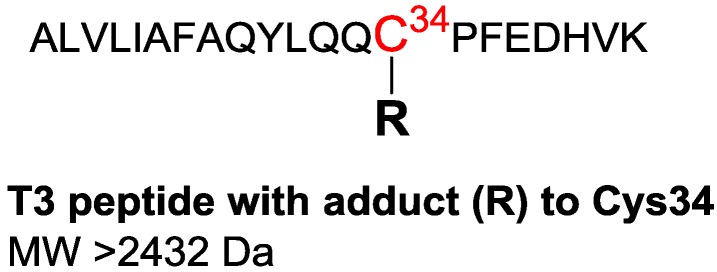
Following tryptic digestion, Cys34 is located on the third largest tryptic peptide, designated T3.

The method for HSA adductomics uses only 5 µL of plasma/serum. The first step in the sample preparation involves precipitation of non-HSA proteins with 60% methanol. Following centrifugation, the protein content of the supernatant is approximately 70% HSA (of total protein content) [36]. A portion of the supernatant is diluted with buffer and digested with trypsin, using a pressurized system (Barozyme HT48 from Pressure Biosciences Inc., South Easton, MA, USA) that facilitates complete digestion in 30 min, without prior reduction of the intramolecular HSA disulfides. The Cys34 residue is located on the third largest tryptic peptide, designated T3 with sequence ^21^ALVLIAFAQYLQQC^34^PFEDHVK^41^ with MW 2432.2562 Da (Figure 1). After digestion, formic acid is added to the sample to prevent further degradation. Prior to LC-MS analysis, a portion of the digest is diluted with 2% ACN/0.2% formic acid, and an isotopically labelled T3 peptide is added as an internal standard to monitor retention time drift and mass accuracy. Quantitation of adducts is performed relative to a housekeeping (HK) peptide, which is an HSA peptide of sequence ^41^LVNEVTEFAK^50^ with MW 1148.6077 Da. The peak area ratio (PAR = adduct peak area/HK peptide peak area) has been shown to be a reliable measure of adduct concentration over a wide dynamic range [36]. Recently the method has been adapted for analysis of HSA extracted from dried blood spots (DBS), which require additional steps prior to digestion [38]. The small amount of protein needed for HSA adductomics, as well as the possibility to use DBS for the analysis, is an attractive advantage of this methodology. The fast sample preparation allows for processing and MS analysis of about 12 samples per day (with duplicate injections).

**Figure 2:**
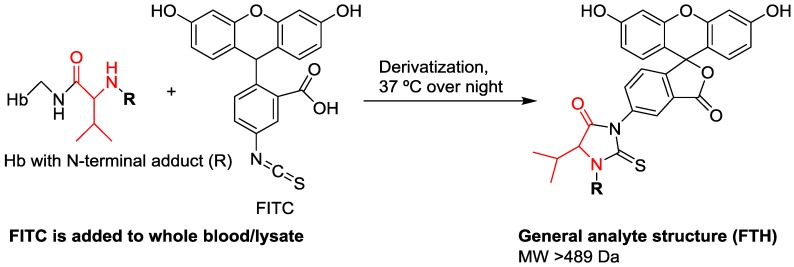
Adducted N-terminal amino acids are detached using the reagent fluorescein isothiocyanate (FITC) generating adduct derivatives in the form of fluorescein thiohydantoins (FTHs).

The methodology for Hb adductomics is based on a modified Edman procedure in which adducted N-terminal amino acids are specifically detached using the reagent fluorescein isothiocyanate (FITC) (Figure 2). In the 1980s, during the development of the first successful method for analysis of N-terminal Hb adducts it was found that the detachment of adducted N-terminal Val with isothiocyanate reagents was favored over non-adducted Val, providing a basis for selective enrichment of adducts [39,40,41]. This methodology was then developed for LC-MS analysis using FITC as the reagent [42,43]. To perform Hb adductomics 5 mg of FITC (dissolved in dimethyl sulfoxide) is added to 250 µL of whole blood or lysate (both work equally well for the methodology) and mixed at 37 °C overnight [44]. This reaction, an example of a modified Edman degradation, generates detached adduct derivatives, in the form of fluorescein thiohydantoins (FTHs) (Figure 2). To purify the FTH derivatives, the proteins are first precipitated with acetonitrile (ACN) and then centrifuged. At this step, stable isotope-substituted internal standards corresponding to one or several FTH derivatives of known adducts are added. The FTH derivatives have a carboxylic acid functional group (originating from the fluorescein part of the molecules), which may be protonated/deprotonated with acids/bases. This property is utilized in the work-up of the FTHs. Following precipitation and centrifugation, the FTH-containing ACN supernatant is alkalized (deprotonation of the carboxylic acid) with ammonium hydroxide then added to a mixed-mode anion exchange solid phase extraction (SPE) column. The deprotonated anionic FTHs are retained on the column, which are then washed with ACN, H_2_O, and cyanoacetic acid in H_2_O, in that order. To elute the FTHs a solution of cyanoacetic acid in ACN is used, which breaks both the hydrophobic and anionic interactions with the column. The eluted samples are evaporated to dryness using air and then re-dissolved in H_2_O/ACN (6:4), preparing the samples for LC-MS analysis. Although the sample processing for Hb adductomics is more time-consuming than the method for HSA adductomics, with the over-night derivatization being the limiting step, the method is fast compared to earlier methods for Hb adduct analysis [40,45] and is applicable for semi-high throughput analysis [44,46].

## 4. Mass Spectrometry

The method for HSA adductomics uses nanoflow LC and nanoelectrospray-ionization and the mass spectrometry is performed with a Hybrid Ion Trap Orbitrap high-resolution mass spectrometer (HRMS) (ThermoFisher Scientific, MA, USA) [36]. One microliter of each sample digest is injected into a monolithic nanoflow column that has excellent properties for separation of hydrophobic peptides and proteins. The analysis requires about 40 min per injection, and each sample is injected in duplicate followed by blank and wash injections, making the total time per sample about 2 h. The ionization is done in positive-ion mode and the mass spectrometer is operated in the data-dependent acquisition (DDA) mode, which selects the most abundant ions for fragmentation (MS2) using the ion trap. The combination of high resolution MS1 data from the Orbitrap analysis and ion trap MS2 data, enables the classification of unknown compounds as adducts and determination of adduct masses with high accuracy.

**Figure 3:**
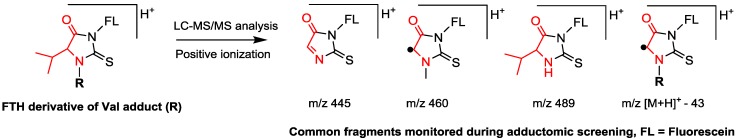
Fluorescein thiohydantoin (FTH) derivatives of N-terminal Hb adducts exhibit similar fragmentation pathways when performing tandem mass spectrometry (MS/MS).

Hemoglobin adductomics has almost exclusively been performed with triple quadrupole mass spectrometers which require a different strategy for untargeted screening of adducts compared to high resolution mass spectrometers. Similar to early methods for DNA adductomics and HSA adductomics [17,18], the screening of N-terminal Hb adducts using triple quadrupole MS has been done in the multiple reaction monitoring (MRM) mode [19]. From the analysis of FTH derivatives of known N-terminal Hb adducts it was observed that all such analytes exhibit similar fragmentation pathways when performing MS/MS, resulting in at least three common fragments (Figure 3). This observation is the underlying principle for MRM screening of FTH derivatives of unknown N-terminal Hb adducts, in which sequential lists of MRM transitions are set up for the m/z range of interest with four diagnostic fragments, corresponding to the most common fragmentation pathways, being screened for each incremental m/z unit. Although the most sensitive alternative on a triple quadrupole platform, the analysis of large numbers of MRM transitions is relatively slow. To acquire a sufficient number of data points for quantitative analysis each sample needs to be injected several times, covering different increments of the studied m/z range with each injection. In the first application of Hb adductomics each sample was injected 12 times to cover a range of 135 m/z, demonstrating the time-consuming aspects of this methodology [19]. Recently, the methodology has been adapted for HRMS using a Hybrid Quadrupole Orbitrap MS (Thermo Scientific, MA, USA) [37]. On this platform the MS analysis was performed in the data independent acquisition (DIA) mode. In this mode, all ions within a specified m/z range are fragmented, with collective fragmentation patterns for all ions within that range recorded. This is an advantageous approach when screening for low-level compounds, such as adducts, in complex biological samples compared to DDA for which only abundant ions will be fragmented, thereby risking missing interesting compounds. In a blood sample derivatized with FITC the concentration of reagent byproducts is high (5 mg FITC is used for each 250 µL of blood; see Sample Preparation above), making DIA a better suited alternative to DDA to maximize detection of potential adducts. A disadvantage with DIA is that the measurements are slower than for DDA, meaning that the samples need to be injected several times to cover large m/z ranges. When screening the m/z range 500–700 m/z for Hb adducts each sample was injected four times, with each injection covering 50 m/z [37]. To confirm possible adducts observed in the DIA mode, additional follow-up measurements were performed in the parallel reaction monitoring mode, in which ions of specific m/z (unit resolution) are fragmented.

For Hb adductomics conventional LC-MS has been performed using a flow rate of 0.12 mL/min and electrospray ionization in the positive mode [19]. A volume of 10–20 µL of the sample is injected (depending on type of instrumentation and purpose of experiment) on a reversed phase C18 column with the dimensions 2.1 mm × 150 mm and a gradient is applied to separate sample components. The chromatographic program is 35 min per injection.

## 5. Data Evaluation

Untargeted screening MS experiments generate huge and complex sets of data, that are both difficult and time-consuming to evaluate. In comparison with metabolomics and proteomics, adductomics is a small field of research and there are no commercial software solutions for the evaluation of adductomic data.

For HSA adductomics, unknown species detected as triply charged ions in the HRMS are classified as Cys34 HSA adducts if they exhibit a sufficient number of *b*^+^- and *y*^2+^-fragments that are characteristic of the T3 peptide (m/z = 811.7594). This process is described in detail by Grigoryan et al. 2016 [36]. To facilitate this time-consuming task, in-house software, written in the programming language R, has been developed for processing MS2 spectra according to the established rules for adduct selection [47]. The internal standard that is added in the final step of sample preparation (isotopically labeled T3 modified with iodoacetamide) is used to monitor variations in retention times and mass accuracy throughout the analysis. The resulting processed spectra are inspected manually to control for false positives. For quantification of detected adducts, selected ion chromatograms generated using the monoisotopic masses (MIMs) of the adducted T3 peptides are used. Peak area ratios relative to the HK peptide facilitate relative quantitation and statistical analyses and can provide approximate concentrations in pmol adduct/mg HSA [36]. The added masses of the adducts are calculated by subtracting the MIM of the thiolate form of the unmodified T3 peptide from the MIM of the adducted T3 peptides. The calculated added masses are used to propose elemental compositions of adducts and to suggest likely structures and electrophilic precursors.

When evaluating data from Hb adductomics, adduct candidates are selected based on the observation of at least two of the four screened diagnostic fragments (see Mass Spectrometry Analysis above and Figure 3). Compounds considered as possible adducts are further studied in product ion scan mode and their fragmentation patterns compared with those of known adduct analytes. The selection of adducts is done manually. The evaluation of MRM data is time-consuming but rather straightforward, which is not the case for DIA data for which the evaluation requires extensive manual inspection as well as follow-up experiments to select adducts with certainty. To determine the added mass of an adduct, the m/z of the FTH derivative of unmodified Val is subtracted from the m/z of the FTH derivative of the Val adduct. Adduct levels of Hb adducts are estimated using a semiquantitative approach, assuming that all FTH derivatives of Val adducts have similar responses in the MS analysis. Internal standard calibration has been done using the stable-isotope internal standard corresponding to the FTH derivative of the Val adduct of acrylamide (AA), which is added to all samples during the sample preparation immediately after the FITC derivatization. A calibration curve of a reference standard of AA-Val-FTH and the corresponding internal standard is used for this purpose. The average of the integrated peak areas for the detected diagnostic transitions for each adduct is used for the determinations. The adduct levels are then adjusted for the Hb concentrations in the blood samples, measured separately using a spectrophotometric device. Adduct levels are reported in the unit pmol/g Hb. The assumption that all FTH derivatives of Val adducts have similar responses in the MS analysis is a simplification but a reasonable approximation for the low molecular weight adducts observed. The supposed similar responses are supported by the comparable responses for the different known adducts that have been studied as FTH derivatives in more detail [48,49,50,51,52].

## 6. Identification of Adducts

Following untargeted detection of adducts the next challenge is their identification. This section describes how previously unknown adducts have been identified following HSA and Hb adductomics. With HSA adductomics about 75 adducts (or HSA variants) have been reported thus far with added masses ranging from −46 to 510 Da [36,53,54,55] (negative added masses refer to truncations and deletions). Most of these adducts have been annotated to presumed structures and 11 have been identified through comparisons with reference standards [36,55,56]. The HSA adducts detected in the four published adductomics studies are presented in Table 3. Annotations include Cys34 oxidation products, truncations and deletions, and disulfides of low-molecular-weight thiols. Many of the HSA modifications detected through adductomics result from oxidation of Cys34 to produce adducts with one, two or three oxygens. Because the one-oxygen addition product, i.e. the sulfenic acid (Cys34-SOH) is unstable, it reacts with the adjacent amino acid Gln33 to produce a cyclic sulfinamide, which is detected [56]. Formation of the Cys34 sulfenic acid, also leads to disulfide formation with small circulating thiols, which represent the largest class of adducts detected with HSA adductomics thus far. With Hb adductomics 25 different N-terminal Val adducts have been detected, with added masses ranging from 15 to 198 Da, with 13 of them identified thus far [19,50,52,57]. These Hb adducts are presented in Table 4.

The results of an adductomic experiment will be a list of adducts detected, with their molecular weights, estimated concentrations, and retention times. This is the only information available to formulate hypotheses about adduct identities. The added mass may be calculated as described above, by subtracting the m/z of an unmodified precursor molecule, from the adduct m/z. The accuracy of the calculated added mass will depend on the type of mass spectrometer used, with high resolution instruments providing more accurate data. Based on the added mass the elemental composition of the adduct may be suggested. A practical tool to help with this task is the Molecular Formula finder, provided by ChemCalc (http://www.chemcalc.org/mf_finder) [58]. With a likely composition of the adduct, the adduct structure or corresponding precursor electrophiles can be suggested. As described above, electrophiles that form adducts commonly have reactive functional groups, such as activated double bonds, aldehydes, and epoxides. Such features should be considered when considering possible precursors, as well as the chemistry of the specific nucleophilic sites and limitations of the methodology used. One limitation with the present method for Hb adductomics is when the N-terminal nitrogen atom is blocked for the reaction with the isothiocyanate reagent, as when it is tertiary or when it is substituted with acyl groups. This exclude ring-closed adducts, e.g., the adduct from diepoxybutane [59], from being detected. Adducts from isocyanates, carbamoylated N-terminal valines, could though be detached as hydantoins from Hb after acidification in a procedure similar to the normal Edman procedure, as has been shown in several studies (e.g., for methylisocyanate [60] and dimethylformamide [61]). Of the identified or annotated adducts observed to Hb and HSA there has been very little overlap thus far (when analyzing blood samples from smokers, acrylonitrile, and ethylene oxide adducts were observed both to Hb and HSA [19,36], and the recently identified 4-hydroxybenzyl Hb adduct may correspond to the unknown HSA adduct with the same annotated composition C_7_H_7_O, added mass 107.0481 Da [52]). This emphasizes the need of several nucleophilic targets when exploring the adductome.

With adducts of low mass there may be few possible structures and precursors, facilitating the adduct identification. However, as MWs exceed about 70 Da the numbers of possibilities increase rapidly, and annotation becomes difficult. To facilitate hypotheses about possible annotations specialized databases can be used. The simplest of these databases is “Search for Species Data by Molecular Weight” provided by the National Institute of Standards and Technologies (https://webbook.nist.gov/chemistry/mw-ser) [62], which list species based on the input value. Although there is no specific database for adductomics, the UNIMOD database that lists protein modifications may be useful (www.unimod.org) [63]. Databases for metabolomics may also contain valuable information. Among these there are large databases such as the human metabolome database (http://www.hmdb.ca/metabolites) [64], but also more specific databases such as the Toxic Exposome Database (T3DB) (http://www.t3db.ca) [65] and the Exposome-Explorer Database (http://exposome-explorer.iarc.fr) [66] can be relevant for adductomics [67]. Having established one or more candidate annotations for a given adduct, the next step is to perform literature searches, consider possible metabolic activations of possible precursors, working backwards to determine whether suspected adduct precursors have been previously described.

Both Hb and HSA adductomics are performed with gradient-elution reversed-phase chromatography that separates compounds based on lipophilicity. Thus, the retention times of the analytes correlate with their lipophilicities, and this information can also be useful in suggesting adduct identities. For FTH derivatives of Val adducts the theoretical Log P values (calculated using ChemDraw from CambridgeSoft, Cambridge, MA, USA) correlate with their retention times (with a coefficient of determination, *R*^2^ = 0.88) [57]. By drawing the hypothetical adduct derivatives in ChemDraw and comparing the theoretical Log Ps with the observed retention times of the investigated unknown adducts, the hypotheses may be further evaluated prior to initiating the costly and time-consuming identification. For the FTH derivatives with relatively low MWs generally ranging from about 500–700 Da, this is a very viable strategy while the situation for the larger T3 adducts (MWs > 2432 Da) is more complex. For such large molecules the retention times are not as affected by small modifications and cannot be expected to be as reproducible as for reverse phase separations of small molecules. Although the chromatographic program includes a 30 min gradient (from 2–45% ACN), the majority of T3 adducts elute during a 5 min period, indicating the small differences in retention even for large modifications such as glutathione (added mass of 306 Da) [36].

**Figure 4:**
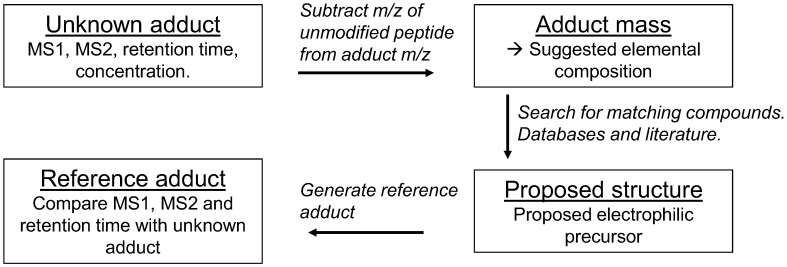
Flowchart depicting the process of identifying unknown adducts using adductome data.

To confirm adduct identities, reference adducts should be synthesized and compared with the unknown adducts of interest. Generation of reference adducts can simply involve adding the proposed precursor electrophiles to either plasma or whole blood/lysate. The concentrations of the electrophiles and the reaction times necessary to yield sufficient concentrations of the adducts vary depending on the reactivity and stability of the electrophiles. Detailed experiments for generating reference adducts have previously been published by Grigoryan et al. [36,55,56] for HSA adducts, and Carlsson et al. [19,57] for Hb adducts. Following incubations with electrophiles the samples should be processed and analyzed according to the respective methods. The synthetic adducts should then be compared with the unidentified adducts observed concurrently in untreated human samples to minimize methodological and instrumental variations. The comparison should be done with regard to m/z of the precursor ions, fragmentation patterns (MS2) as well as retention times. The identification process is summarized in the form of a flowchart in Figure 4.

The small FTH derivatives of Hb-Val adducts are advantageous for identification purposes, with highly reproducible retention times and distinct MS2 spectra in which the relative abundances of fragments may be used to match spectra for unknown and reference adducts with high agreement [50,57]. For example, the electrophiles methylglyoxal and acrylic acid form Hb adducts with the same elemental composition, assumed to be 1-carboxyethyl-Val, and 2-carboxyethyl-Val respectively, but the retention times and MS2 spectra of their FTH derivatives are significantly different and allows for unambiguous identification [57]. This simpler pathway of generating reference adducts does not mean that a fully characterized (with NMR) reference compound is not often desired, and sometimes required for unequivocal identification.

The identification of adducts is complicated and time-consuming, and potentially expensive. In many cases there will be structural isomers that will complicate structural identification. For some small adducts, such as addition of one, two or three oxygens to Cys34, accurate mass is sufficient to confirm the identity. However, in most cases the adducts and often precursor molecules will require regular organic synthesis work. A recent example, representing a chemically complex generation of a reference adduct is the identification of 4-hydroxybenzyl adducts to N-terminal Val in Hb [52]. Two precursors were found to generate the adduct; 4-quinone methide and 4-hydroxybenzaldehyde. The quinone methide is not commercially available and not chemically stable. A precursor with protective groups was therefore synthesized in-lab, and activated prior to the reaction with Val. This reflect the often complex process to generate a reference adduct.

## 7. Conclusions

The concept of protein adductomics was introduced nearly a decade ago. Since then the methodologies have been refined for the current generation of instrumentation and adapted to showcase the strengths and possibilities of adductomics. Many previously unknown adducts have been detected, and many of them have been identified. When comparing the present methodologies for HSA and Hb adductomics, the HSA method is more versatile and suited for high-throughput studies. By analyzing tryptic digests of HSA this method is truly untargeted and although adducts to Cys34 have been the primary focus to date, adducts to other nucleophilic sites in HSA (e.g., Lys199) may be detected simultaneously in a single LC-MS run. Other advantages of HSA adductomics is the low sample volumes needed (5 µL) and the streamlined selection of adducts using the in-house software. The method for Hb adductomics is limited to N-terminal Hb adducts and require relatively large sample volumes (250 µL), and will benefit from lower detection levels with more sensitive MS instrumentation than used in the published adductomics work. This methodology, however, benefits from the relatively small molecules resulting from the derivatization of N-terminal adducts, that are advantageous in the identification of unknown adducts. The FTH derivatives separate well using reversed phase chromatography, with highly reproducible retention times, and give distinct MS2 spectra. The minimal overlap of the adducts so far detected and identified with the different methods highlights the need to analyze several nucleophilic sites to explore the full adductome. In the small field of adductomics the two methods described in this review are the two best suited methods for analyzing large number of samples, even though the numbers of samples that can be analyzed during a certain period are limited compared to methods used, for example, for metabolomics.

The constant improvement of MS instrumentation will benefit adductomics by decreasing detection limits and improving mass accuracies. The most challenging aspect of adductomics is, however, the quantification of unknown adducts. Hemoglobin adductomics rely on a more traditional approach with internal standard calibration and report estimated adducts levels assuming similar responses for all adducts. Human serum albumin adductomics employs an innovative housekeeping peptide to normalize peak areas for the amounts of HSA in the digests. Both approaches acknowledge the impossible task of giving accurate quantitative estimates of unknowns, and for the purposes of adductomics they work well, meaning that the relative concentrations of a set of adducts in a population of samples can be obtained for preliminary evaluations. If follow-up studies heighten interest in these adducts, then they can be identified and standardized for subsequent targeted analyses. Another challenging aspect of adductomics is formation of artifacts from storing and processing of samples. In the case of HSA adductomics the digestion procedure could potentially expose the nucleophilic sites in the protein for electrophiles present in the samples. For Hb adductomics the derivatization procedure could potentially form byproducts or chemically affect adducts. To control for such processes, various control experiments may be performed (cf. Carlsson 2014 [19]), but it is not likely that artefact formation for certain adducts, for instance during storage [68], may be fully controlled. Thankfully, it can be assumed that the extent of such artifact formation should be the same for all samples processed in a given study, e.g., from disease cases and concurrent controls, thereby reducing the likelihood of false positives in the statistical analysis.

As a complement to the HSA adductomics method we are currently investigating the analysis of intact HSA isoforms in plasma samples, using a time-of-flight MS. When analyzing the intact protein, the sample preparation prior to the MS analysis is minimal, as is the risk of introducing artifacts. Cysteine 34 of HSA is easily oxidized and the analysis of the intact protein provides valuable insights about the state of HSA in samples, prior to digestion. By comparing the two approaches, using both bottom-up and top-down proteomic data of HSA in plasma samples, a more complete view of HSA and the various modified forms is gained. An important goal of adductomics is to provide a deeper understanding of the underlying chemistry describing how proteins are modified by reactive compounds in vivo. With the methods described in this review we have increased our understanding of this fascinating aspect of biological chemistry.

## Figures and Tables

**Table 1:** Properties of hemoglobin and human serum albumin.

Property	Human Hemoglobin	Human Serum Albumin
Molecular weight	Tetramer, 64 kDa. Composed of two α- and two β-subunits (in adult Hb), both weighing 16 kDa	67 kDa
Main functions	Oxygen transport	Transport of e.g., hormones and fatty acids, maintains oncotic pressure, antioxidant.
Localization	Red blood cells	Blood plasma
Conc (mg/mL)	120–160 (in blood)	35–55 (in serum)
Turnover rate (days)	~120 (lifespan of red blood cells)	~20 (half-life in serum)

**Table 2:** Critical aspects of the methodologies for Hb and HSA adductomics.

Methodological Aspect	Hb Adductomics	HSA Adductomics
Nucleophilic site	N-terminal Val	Cys34
Sample volume	250 µL whole blood/lysate	5 µL plasma/serum
Enrichment of adducts	Detachment of adducts using the reagent fluorescein isothiocyanate, followed by clean-up using solid phase extraction	Precipitation of non-HSA proteins, followed by tryptic digestion
Analytes	Fluorescein thiohydantoin derivatives of adducted Val	Tryptic peptide containing the adducted Cys34 residue
MW of analytes	>502 Da (starting at the MW of the methyl adduct)	>2432 Da (analyzed as triply charged positive ions)
Analytical method	Liquid chromatography-mass spectrometry (LC-MS)/high-resolution MS (LC-HRMS)	Nano-liquid chromatography-high-resolution mass spectrometry (nLC-HRMS)
Type of LC column	Reversed phase, C18, 120 µL/min	Monolithic, 750 nL/min
Type of MS and MS method	MS: Triple quadropole in multiple reaction monitoring mode; HRMS: Orbitrap in data independent acquisition mode	HRMS: Orbitrap in data dependent acquisition mode

**Table 3:** HSA adducts detected on the T3 peptide that contain Cys34.

Adduct	Ret. Time (min)	MIM Observed (m/z, +3)	Added Mass (Da)	Suggested Elemental Composition of Added Mass (to Cys34S^−^)	Annotation
796.43 ^a,b,d^	27.5	796.4309	−45.9913	−CH_2_S	Cys34→Gly
800.43 ^a,b,d^	28.4	800.4317	−33.9873	−SH_2_	Cys34→Dehydroalanine
805.76 ^a,b,d,e^	27.2	805.7632	−17.9965	−SH_2_, +O	Cys34→Oxoalanine
808.73 ^a,b,c,d^	28.3	808.7306	−9.0923		Not Cys34 adduct
810.45 ^a,c^	30.5	810.4536	−3.9280		Not Cys34 adduct
811.43 ^a,b,c,d^	30.4	811.4254	2431.2480	+C_114_H_172_N_27_O_30_S	T3 Dimer ^f^
811.76_1 ^a,b,c,d^	27.9	811.7608	1.0072	+H	T3 Labile adduct
811.76_2 ^a,b,c,d,e^	28.6	811.7609	1.0097	+H	Unmodified T3 ^f^
816.42 ^a,b,c,d,e^	27.8	816.4200	13.9766	−H_2_, +O	S-Monooxidation ^f^
816.43 ^a,b,c,d,e^	29.1	816.4321	15.0233	+CH_3_	Methylation (not at Cys34)
820.09 ^a,c,d^	28.8	820.0920	25.9995	+CN	S-Cyanylation
822.42 ^a,b,c,d,e^	27.7	822.4236	32.9946	+HO_2_	S-Dioxidation ^f^
823.39 ^a^	27.2	823.3971	35.9173		Not Cys34 adduct
825.76 ^d^	26.7	825.7644	43.0188	+C_2_H_3_O	S-Acetylation
826.10 ^d^	26.7	826.0990	44.0226	+C_2_H_4_O	Unknown (likely S-addition of an aldehyde)
826.44 ^a^	27.9	826.4358	45.0332	+C_2_H_5_O	Ethylene oxide adduct
827.09 ^b,d^	28.8	827.0901	46.9960	+CH_3_S	S-Methylthiolation
827.09 ^c^	30.1	827.0946	47.0022	+HO_2_ + CH_2_	Cys34 sulfinic acid plus methylation (not Cys34)
827.1 ^d^	27.5	827.0958	47.0129	+CH_3_O_2_	S-(O)-O-CH3
827.76 ^a,b,c,d,e^	28.0	827.7550	48.9889	+HO_3_	S-Trioxidation ^f^
829.13 ^a^	27.4	829.1264	53.1052		Not Cys34 adduct
829.40 ^a,b^	28.6	829.3972	53.9177		Not Cys34 adduct
829.44 ^a^	28.9	829.4369	54.0366	+C_3_H_4_N	Acrylonitrile adduct
830.41 ^d^	26.5	830.4059	56.9433		Unknown
830.44 ^d^	27.3	830.4359	57.0333	+C_3_H_5_O	Unknown (likely S-addition of an aldehyde)
830.77 ^a^	27.5	830.7685	58.0313	+C_2_H_4_NO	Methylisocyanate adduct
833.08 ^d^	27.8	833.0813	64.9696	+HO_2_S	S-Addition of SO_2_
835.11 ^a, d^	28.2	835.1079	71.0494	+C_4_H_7_O	S-Addition of crotonaldehyde ^f^
837.10 ^d,e^	27.4	837.1041	77.0380	+C_6_H_5_	S-Phenylation
839.78 ^d,e^	27.7	839.7797	85.0647	+C_5_H_9_O	S-Addition of tiglic aldehyde ^f^
841.10 ^b,c^	28.5	841.0987	89.0183	+C_3_H_5_O_3_	S-Addition of pyruvate or malonate semialdehyde
841.43 ^a^	27.8	841.4251	90.0013	+C_2_H_4_NOS	S-Mercaptoacetamide
841.75 ^a,b,c,d^	28.6	841.7529	90.9827	+C_2_H_3_O_2_S	S-Addition of mercaptoacetic acid
845.42 ^a,b,c,d^	27.7	845.4249	101.9987	+C_3_H_4_NOS	S-Addition of Cys (-H_2_O)
845.75 ^b^	28.6	845.7528	102.9842	+C_3_H_3_O_2_S	S-Cys (possibly NH_2_ → OH, −H_2_O)
847.10 ^d^	26.7	847.0963	107.0145	+C_3_H_7_O_2_S	S-Methylethyl-sulfonylation
847.11 ^a,b,c^	30.2	847.1082	107.0481	+C_7_H_7_O	S-Addition of benzaldehyde or quinone methide
847.77 ^a,b^	27.3	847.7664	109.0251		Cys34 Adduct with unknown annotation
849.07 ^a,b,d^	28.0	849.0698	112.9353	+HO_3_S_2_	S-Addition of S_2_O_3_H
850.10 ^a,b,d^	27.9	850.0975	116.0182	+C_4_H_6_NOS	S-Addition of hCys (-H_2_O)
851.43 ^a,b,c,d^	26.9	851.4290	120.0109	+C_3_H_6_NO_2_S	S-Addition of Cys ^f^
851.76 ^a,b,c,d^	27.8	851.7572	120.9973	+C_3_H_5_O_3_S	S-Addition of Cys (NH_2_→OH)
853.78 ^a,b^	27.7	853.7839	127.0776		Cys34 Adduct with unknown annotation
854.44 ^d^	25.9	854.4428	129.0539	+C_6_H_9_O_3_	S-Addition of BDE
855.44 ^b^	29.0	855.4373	132.0378	+C_8_H_6_NO	Oxindole
856.10_1 ^a,b,c,d^	27.0	856.1003	134.0250	+C_4_H_8_NO_2_S	S-Addition of hCys ^f^
856.10_2 ^a,b,c,d^	27.3	856.1001	134.0244	+C_4_H_8_NO_2_S	S-Addition of hCys ^f^
856.43 ^d^	27.2	856.4287	135.0117	+C_4_H_7_O_3_S	S-Addition of hCys (NH_2_→OH)
857.09 ^c^	29.0	857.0876	136.9812		Unknown
857.1 ^a,b,d^	27.4	857.1000	137.0257		Unknown
859.41 ^d^	25.5	859.4085	143.9513		Unknown
860.77 ^b,c^	29.3	860.7717	148.0342	+C_4_H_8_NO_2_S + CH_2_	S-hCys, plus methylation (not Cys34)
862.09 ^a,d^	26.3	862.0881	151.9901		Not Cys34 adduct
864.08 ^a,b,d^	26.2	864.0772	157.9574		Not Cys34 adduct
864.43 ^a,b,c,d^	27.5	864.4319	159.0198	+C_5_H_7_N_2_O_2_S	S-Addition of CysGly (-H_2_O)
865.43 ^a,b,c^	28.3	865.4314	162.0176	+C_5_H_8_NO_3_S	S-(N-acetyl)Cys
866.76 ^b^	27.0	866.7572	165.9973	+C_4_H_8_NO_2_S_2_	S-S-hCys trisulfide
870.44 ^a,b,c,d^	26.5	870.4360	177.0319	+C_5_H_9_N_2_O_3_S	S-Addition of CysGly ^f^
872.7309 ^a^	26.7	872.7309	183.9187		Not Cys34 adduct
870.44 ^a,b,d^	27.2	873.4310	186.0187		Unknown
875.11 ^b,c^	27.5	875.1062	191.0409	+C_5_H_9_N_2_O_3_S + CH_2_	S-CysGly, plus methylation (not Cys34)
875.42 ^b^	27.7	875.4231	191.9950		Cys34 Adduct with unknown annotation
894.13 ^b,c^	26.1	894.1270	248.1029		Unknown
894.44 ^a,b,c,d^	27.0	894.4426	249.0516	+C_8_H_13_N_2_O_5_S	S-Addition of GluCys ^f^
899.11 ^c^	29.3	899.1140	263.0612		Unknown
913.45 ^a,b,c,d^	26.9	913.4494	306.0722	+C_10_H_16_N_3_O_6_S	S-Addition of GSH ^f^
918.12 ^c^	29.2	918.1222	320.0852		Unknown
927.14 ^c^	27.1	927.1408	347.1412		Unknown
928.78 ^c^	32.2	928.7842	352.0712		Unknown
931.82 ^b,c,d^	25.9	931.8215	361.1881		Unknown
941.16 ^b^	25.3	941.1570	389.1967		Unclear modification site
965.49 ^b,c,d^	25.8	965.4920	462.1994		Unknown
970.16 ^c^	28.1	970.1643	476.2112		Unknown ^c^
974.51 ^b,d^	25.1	974.5068	489.2460		Unknown
976.82 ^b,c^	28.0	976.8205	496.1840		Unknown
981.50 ^b^	25.3	981.4956	510.2126		Cys34 adduct with unknown annotation

Notes: GSH, glutathione; hCys, homocysteine; MIM, monoisotopic mass. ^a^ Detected by Grigoryan et al. 2016 [36]. ^b^ Detected by Lu et al. 2017 [53].^c^ Detected by Liu et al. 2018 [54]. ^d^ Detected by Grigoryan et al. 2018 [55]. ^e^ Adduct was detected in samples that were reduced with tris(2-carboxyethyl)phosphine (TCEP) prior to digestion; reduces all disulfide bonds. ^f^ Annotation confirmed with a synthetic standard.

**Table 4:** N-terminal Hb adducts detected using adductomics to date.

[M+H]^+^ m/z	Identity/Precursor ^a^	rt (min)	Added Mass (Da) to Val-NH	Elemental Composition
503.1 ^b, c^	**Methylation**	16.6	15	CH_3_
517.1 ^b, c^	**Ethylation**	17.9	29	C_2_H_5_
519.1 ^c^	Unknown	12.3	31	Unknown
519.1 ^c^	Unknown	15.7	31	Unknown
533.1 ^b, c^	**Ethylene oxide**	14.1	45	C_2_H_5_O
542.1 ^b^	**Acrylonitrile ^d^**	18.0	54	C_3_H_4_N
547.1 ^b, c^	**Carboxy-methylation/Glyoxal**	14.2	59	C_2_H_3_O_2_
559.1 ^b, c^	**Methyl vinyl ketone**	16.6	71	C_4_H_7_O
560.1 ^b, c^	**Acrylamide**	12.9	72	C_3_H_6_NO
561.1 ^b, c^	**Acrylic acid/Carboxyethylation**	14.5	73	C_3_H_5_O_2_
561.1 ^c^	**Methylglyoxal**	12.6	73	C_3_H_5_O_2_
563.1 ^c^	**Glycidol**	12.4	75	C_3_H_7_O_2_
573.1 ^b, c^	**Ethyl vinyl ketone**	18.1	85	C_5_H_9_O
576.1 ^b^	**Glycidamide**	12.2	88	C_3_H_6_NO_2_
577.1 ^b, c^	Unknown	13.0	89	Unknown
593.1 ^b, c^	Unknown	11.3	105	Unknown
595.1 ^c^	Unknown	15.1	107	Unknown
595.1 ^b,c^	**4-Hydroxybenzyl**	17.00	107	C_7_H_7_O
615.1 ^b,c^	**1-Octen-3-one**	22.2	127	C_8_H_15_O
617.1 ^b,c^	Unknown	14.8	129	Unknown
625.1 ^b,c^	Unknown	13.9	137	Unknown
631.1 ^b,c^	Unknown	15.2	143	Unknown
651.1 ^c^	Unknown	11.2	163	Unknown
659.1 ^c^	Unknown	16.4	171	Unknown
686.1 ^c^	Unknown	11.2	198	Unknown

Notes: ^a^ The names of identified adducts or known/probable precursors are in bold (confirmed with reference adducts). ^b^ Detected by Carlsson et al. 2014 [19]. ^c^ Detected by Carlsson et al. 2017 [37]. ^d^ Acrylonitrile adducts are typically only observed in samples from smokers.

## Abbreviations

AA	acrylamide
ACN	acetonitrile
DIA	data independent acquisition
DDA	data-dependent acquisition
FITC	fluorescein isothiocyanate
FTH	fluorescein thiohydantoin
Hb	hemoglobin
HRMS	high-resolution mass spectrometry
HK	housekeeping peptide
HSA	human serum albumin
LC-MS	liquid chromatography-mass spectrometry
MS	mass spectrometry
MW	molecular weight
MIM	monoisotopic mass
MRM	multiple reaction monitoring
PAR	peak area ratio
RBC	red blood cells
SPE	solid phase extraction
